# Readmission to a surgical intensive care unit: incidence, outcome and risk factors

**DOI:** 10.1186/cc7023

**Published:** 2008-10-06

**Authors:** Axel Kaben, Fabiano Corrêa, Konrad Reinhart, Utz Settmacher, Jan Gummert, Rolf Kalff, Yasser Sakr

**Affiliations:** 1 Department of Anesthesiology and Intensive Care, Friedrich Schiller University Hospital, Erlanger Allee 101, Jena, 07743, Germany; 2 Department of Vascular and General Surgery, Friedrich Schiller University Hospital, Erlanger Allee 101, Jena, 07743, Germany; 3 Department of Cardiothoracic Surgery, Friedrich Schiller University Hospital, Erlanger Allee 101, Jena, 07743, Germany; 4 Department of Neurosurgery, Friedrich Schiller University Hospital, Erlanger Allee 101, Jena, 07743, Germany

## Abstract

**Introduction:**

We investigated the incidence of, outcome from and possible risk factors for readmission to the surgical intensive care unit (ICU) at Friedrich Schiller University Hospital, Jena, Germany.

**Methods:**

We conducted an analysis of prospectively collected data from all patients admitted to the postoperative ICU between September 2004 and July 2006.

**Results:**

Of 3169 patients admitted to the ICU during the study period, 2852 were discharged to the hospital floor and these patients made up the study group (1828 male (64.1%), mean patient age 62 years). The readmission rate was 13.4% (n = 381): 314 (82.4%) were readmitted once, 39 (10.2%) were readmitted twice and 28 (7.3%) were readmitted more than twice. The first readmission to the ICU occurred within a median of seven days (range 5 to 14 days). Patients who were readmitted to the ICU had a higher simplified acute physiology II score (37 +/- 16 versus 33 +/- 16; p < 0.001) and sequential organ failure score (6 +/- 3 versus 5 +/- 3; p = 0.001) on initial admission to the ICU than those who were not readmitted. In-hospital mortality was significantly higher in patients readmitted to the ICU (17.1% versus 2.9%; p < 0.001) than in other patients. In a multivariate analysis, age (odds ratio (OR) = 1.13 per 10 years; 95% confidence interval (CI) = 1.03 to 1.24; p = 0.04), maximum sequential organ failure score (OR = 1.04 per point; 95% CI = 1.01 to 1.08; p = 0.04) and C-reactive protein levels on the day of discharge to the hospital floor (OR = 1.02; 95% CI = 1.01 to 1.04; p = 0.035) were independently associated with a higher risk of readmission to the ICU.

**Conclusions:**

In this group of surgical ICU patients, readmission to the ICU was associated with a more than five-fold increase in hospital mortality. Older age, higher maximum sequential organ failure score and higher C-reactive protein levels on the day of discharge to the hospital floor were independently associated with a higher risk of readmission to the ICU.

## Introduction

Discharge from the intensive care unit (ICU) at the earliest appropriate time reduces excessive and unnecessary use of this expensive health care facility and improves the availability of beds for other critically ill patients requiring ICU admission [1]. However, early discharge of ICU patients to general wards may expose them to inadequate levels of care. Moreover, early discharge may result in ICU readmission during the same hospitalisation with the possibility of a worsening of the patient's original disease process, increased morbidity and mortality rates, a longer length of stay and increased total costs [2-4]. ICU readmission rates reported in the literature vary from 0.9% [5] to 19% [6] with mortality rates for readmitted patients ranging from 26% to 58% [3,4,7,8].

Several studies have attempted to identify predictors of ICU readmission [1-4,8-10]. However, they have been limited by small sample size [3,4,9,11,12], the retrospective nature of data collection [1-6,8,10-16], long study periods [5] and a lack of appropriate multivariate adjustment for possible confounders [4,14]. Furthermore, most of the studies involved patients admitted to mixed medical/surgical ICUs with differences in severity of illness, length of stay, diagnosis and outcomes among these patients [15]. Large multicentre studies have also been performed to investigate the incidence of and risk factors for readmission to the ICU [1,10,17]; however, heterogeneity among contributing centres may limit extrapolation of the results to individual ICUs.

The aim of our study was to investigate the incidence of, outcome from and possible risk factors for readmission in a large cohort of patients in the surgical ICU and to identify predictors of worse outcome in these patients.

## Materials and methods

The study was approved by the institutional review board of Friedrich Schiller University hospital, Jena, Germany, which waived informed consent due to the anonymous and observational nature of the study. All adult patients (older than 18 years) admitted to the surgical ICU of the hospital between September 2004 and July 2006 were included in the analysis.

### Data collection

Data were collected from vital sign monitors, ventilators and infusion pumps, and automatically recorded by a clinical information system (Copra System GmbH, Sasbachwalden, Germany) introduced to the ICU in 1998. The clinical information system provides staff with complete electronic documentation, order entry (eg, medications) and direct access to laboratory results.

The simplified acute physiology score (SAPS) II [18], therapeutic intervention score-28 (TISS-28) [19] and sequential organ failure assessment (SOFA) scores [20] were calculated daily by the attending physician in charge of the patient. SOFAmax was defined as the maximum SOFA score recorded during the ICU stay. Data recorded prospectively on admission also included age, gender, referring facility, primary and secondary admission diagnoses, and surgical procedures before admission. Sepsis syndromes were defined according to consensus conference definitions [21] and were recorded daily by the attending physician in a special section of the clinical information system. Admission diagnosis was categorised retrospectively on the basis of prospectively recorded codes from the *International Classification of Diseases*-10 and electronic patient charts. Comorbidities were defined according to the definitions provided in the original SAPS II paper [18]. For the purpose of this analysis, the following comorbidities were grouped together to reduce the number of covariates in the final multivariate model: metastatic and non-metastatic cancer; type 1 and type 2 diabetes; and chronic renal failure with or without haemodialysis.

Readmission was defined as admission to the ICU of a patient who had previously been admitted to the ICU during the same hospitalisation period. All admission and discharge dates were available from the clinical information system. Planned admission was defined as an admission after elective surgery, which was scheduled 24 hours before the surgical procedure.

### ICU organisation

The ICU at the Friedrich Schiller University hospital is a closed surgical ICU operated by the Department of Anesthesiology and Intensive Care Medicine. A consultant intensivist with a special qualification in intensive care medicine is available in-house 24 hours a day. Attending physicians and in-training residents are available throughout the day (on 12-hour shifts). There is no reduction in personnel or in ICU activities during night shifts or at weekends. Rounds are conducted daily by ICU physicians, nursing staff and the operating surgical team. ICU admission and discharge decisions are made by the consultant intensivist on-duty. Due to the absence of step-down or high-dependency units in the institution, patients are discharged from the ICU only when they are haemodynamically stable with an acceptable general condition and adequate organ function.

### Statistical analysis

Data were analysed using SPSS 13.0 for windows (SPSS Inc, Chicago, IL). The Kolmogorov-Smirnov test was used to verify the normality of distribution of continuous variables. Non-parametric tests of comparison were used for variables evaluated as not being normally distributed. Difference testing between groups was performed using a Wilcoxon test, Mann-Whitney U test, chi-squared test and Fisher's exact test as appropriate. A Bonferroni correction was used for multiple comparisons. A Friedmann test was used to compare the evolution of SOFA scores over time.

We performed a multivariate logistic regression analysis, with readmission to the ICU as the dependent factor, of the overall population. Variables included in the logistic regression analysis were age, gender, comorbid diseases, the source of admission, SAPS II and SOFA scores on admission, SOFAmax, the type of surgery undergone, the presence of sepsis syndromes and parameters of organ function on the day of discharge from the ICU. Colinearity between variables was excluded before modelling. Another multivariate logistic regression analysis was performed to identify risk factors for in-hospital mortality in patients who were readmitted to the ICU. To avoid 'over fitting' of the second model due to the low in-hospital mortality event rate, variables were introduced to this model if significantly associated with a higher risk of in-hospital death on a univariate basis at a p < 0.2.

Continuous data are presented as mean ± standard deviation (sd) and categorical data as number and percentage, unless otherwise indicated. All statistics were two-tailed and a p < 0.05 was considered statistically significant.

## Results

### Study group characteristics

**Figure 1:**
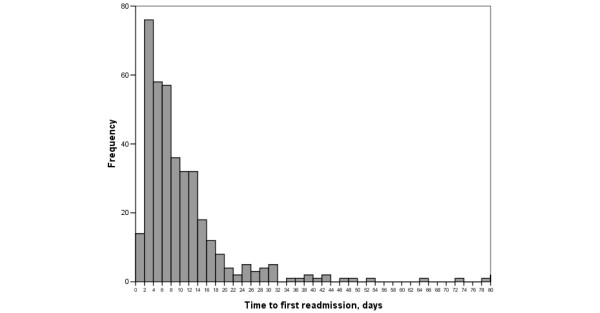
Histogram representing time to first readmission to the intensive care unit (ICU).

Of 3169 patients admitted to the ICU during the study period, 173 (5.5%) died in the ICU and 144 (4.5%) were discharged to other hospitals: 2852 patients were discharged to the hospital floor and those patients made up the study group (1828 male (64.1%), mean patient age 62 years). The readmission rate was 13.4% (n = 381): 314 (82.4%) were readmitted once, 39 (10.2%) were readmitted twice and 28 (7.3%) were readmitted more than twice, giving a total of 476 readmission episodes. The first readmission to the ICU occurred within a median of seven days (range = 5 to 14 days) (Figure 1). The characteristics of the study group are presented in Table 1.

**Table 1:** Characteristics of the study groups on admission to the intensive care unit (ICU).

	All patients (n = 2852)	No readmission (n = 2471)	Readmission (n = 381)	p value
Age, mean ± SD (years)	62 ± 15	61 ± 15	64 ± 14	0.001

Male gender (%)	1828 (64.1)	1578 (63.9)	250 (65.6)	0.506

Source of admission (%)				< 0.001

Operating room	2213 (77.6)	1944 (78.7)	269 (70.6)	

Emergency room	130 (4.6)	110 (4.5)	20 (5.2)	

Other hospital	169 (5.9)	136 (5.5)	33 (8.7)	

Others	172 (6.0)	133 (4.8)	39 (10.3)	

Comorbidities (%)				

Cancer	628 (22.0)	555 (22.5)	73 (19.2)	0.148

Cancer therapy	61 (2.1)	52 (2.1)	9 (2.4)	0.746

Haematological cancer	6 (0.2)	6 (0.2)	-	1.000

Chronic heart failure (NYHA IV)	48 (1.7)	38 (1.5)	10 (2.6)	0.125

Cirrhosis	65 (2.3)	55 (2.2)	10 (2.6)	0.627

Hypertension	1437 (50.4)	1247 (50.5)	190 (49.9)	0.828

Chronic renal failure	288 (10.1)	240 (9.7)	48 (12.5)	0.036

Diabetes	617 (21.6)	522 (21.1)	95 (24.9)	0.137

Primary diagnosis (%)				0.024

Planned postoperative	2268 (79.5)	1995 (80.7)	273 (71.7)	

Unplanned admissions*				

Trauma	139 (4.9)	122 (4.9)	17 (4.5)	

Cardiovascular	124 (4.3)	92 (3.7)	32 (8.4)	

Neurological	109 (3.8)	93 (3.8)	16 (4.2)	

Gastrointestinal	64 (2.2)	49 (2.0)	15 (3.9)	

Respiratory	30 (1.1)	23 (0.9)	7 (1.8)	

Others	116 (4.1)	95 (3.8)	21 (5.5)	

Sepsis syndromes (%)				0.018

SIRS	642 (22.5)	552 (22.3)	90 (23.6)	

Sepsis	57 (2.0)	45 (1.8)	12 (3.1)	

Severe sepsis/septic shock	32 (1.1)	23 (0.9)	9 (2.3)	

Surgery within 24 hours of admission (%)	2412 (84.6)	2113 (85.5)	299 (87.5)	< 0.001

Cardiac surgery	1061 (37.2)	933 (37.8)	128 (33.6)	0.118

Gastrointestinal	564 (19.8)	486 (19.7)	78 (20.5)	0.714

Neurosurgery	415 (14.6)	361 (14.6)	54 (14.2)	0.822

Trauma	169 (5.9)	149 (6.0)	20 (5.2)	0.548

Thoracic surgery	156 (5.5)	138 (5.6)	18 (4.7)	0.492

Others**	104 (3.6)	98 (3.9)	7 (1.8)	0.123

Mechanical ventilation	1339 (49.2)	1155 (48.9)	184 (50.9)	0.503

Admission scores, mean ± SD				

TISS-28 score	41.8 ± 10.7	41.7 ± 10.6	42.1 ± 11.3	0.367

SOFA score	5.1 ± 3.4	5.0 ± 3.4	5.7 ± 3.5	0.001

SAPS2 score	33.5 ± 16.4	32.9 ± 16.3	37.1 ± 16.4	< 0.001

* Trauma = monotrauma without brain trauma, polytrauma without brain trauma; cardiovascular = cardiac arrest needing cardiopulmonary resuscitation before ICU admission, shock requiring vasopressor/inotropic drugs, chest pain, arrhythmia, cardiac failure (left, right or global); neurological = coma, stupor, vigilance disturbances, confusion, agitation, delirium, seizures, focal neurological deficit and intracranial mass effect; gastrointestinal = bleeding (gastrointestinal tract), acute abdomen, severe pancreatitis, liver failure; respiratory = acute lung injury and acute respiratory distress syndrome, acute-on-chronic respiratory failure and impaired respiratory function but less than for acute lung injury; renal = pre-renal acute renal failure, obstructive acute renal failure; haematological = haemorrhagic syndrome, disseminating intravascular coagulation; metabolic = acid-base and/or electrolyte disturbance, hypoglycaemia and hyperglycaemia.

** Renal/urinary tract, metabolic, obstetric/gynaecological surgery.

NYHA = New York Heart Association; SD = standard deviation; SIRS = systemic inflammatory response syndrome; SAPS = simplified acute physiology score; SOFA = sequential organ failure assessment; TISS = therapeutic intervention scoring system.

Patients who were readmitted to the ICU were older, had a higher incidence of chronic renal failure and sepsis syndromes, were more likely to be unplanned admissions and had higher SAPS II and SOFA scores on initial admission to the ICU compared with patients who were not readmitted. Patients who were readmitted to the ICU underwent more surgical procedures within 24 hours of the initial admission compared with patients who were not readmitted; however, the incidence of major surgical procedures was similar between the two groups. During the weekends, 917 patients (32.2%) were discharged to the hospital ward and 704 patients (24.7%) were discharged to the hospital ward during the night (8 pm to 8 am). There were no differences in the frequencies of weekend (24.4% versus 26.5%; p = 0.375) or nocturnal discharges (32.6% versus 29.1%; p = 0.175) between patients who were not readmitted and those who were readmitted to the ICU.

### Characteristics of readmissions to the ICU compared with initial admission

Of the 476 readmission episodes, 223 (46.8%) were planned and 253 (53.2%) were unplanned postoperative admissions (Table 2). Cardiovascular and respiratory complications were the most common reasons for unplanned readmissions (14.3% and 13%, respectively). On the day of readmission, cardiac surgery, gastrointestinal surgery and neurosurgery were performed in 18.1%, 18.1% and 12.1% of patients, respectively. Unplanned admissions contributed to 30.2% of the initial admissions to the ICU and to about 60% of the second or third readmissions (Table 2).

**Table 2:** Characteristics of readmissions to the intensive care unit (ICU)

	Readmission episodes (n = 476)	Initial admission (n = 381)	First readmission (n = 381)	Second readmission (n = 67)	Third or more readmission (n = 28)
Primary diagnosis					

Planned postoperative	223 (46.8)	273 (71.7)	185 (48.6)^$^	27 (40.3)^$^	11 (39.3)^$^

Unplanned admissions*	253 (53.2)	108 (28.3)	196 (51.4)^$^	40 (59.7)^$^	17 (60.7)^$^

Cardiovascular	68 (14.3)	32 (8.4)	57 (15)	9 (13.4)	2 (7.1)

Trauma	-	17 (4.5)	-	-	-

Neurological	29 (6.1)	16 (4.2)	26 (6.8)	1 (1.5)	2 (7.1)

Gastrointestinal	40 (8.4)	15 (3.9)	28 (7.3)	9 (13.4)	3 (10.7)

Respiratory	62 (13.0)	7 (1.8)	46 (12.1)	13 (19.4)	3 (10.7)

Others	54 (11.3)	21 (5.5)	39 (10.2)	8 (12.0)	7 (25.1)

Surgery on the day of admission	280 (58.8)	299 (87.5)	229 (60.1)^$^	34 (50.7)^$^	17 (60.7)^$^

Cardiac surgery	86 (18.1)	128 (33.6)	72 (18.9)	10 (14.9)	4 (14.3)

Gastrointestinal	86 (18.1)	78 (20.5)	59 (15.5)	15 (22.4)	12 (42.9)^$^

Neurosurgery	59 (12.4)	54 (14.2)	55 (14.4)	4 (6.0)	-

Trauma	-	20 (5.2)	-	-	-

Thoracic surgery	37 (7.8)	18 (4.7)	28 (7.3)	7 (10.4)	2 (7.1)

Others**	22 (4.6)	7 (1.8)	21 (5.6)	1 (1.5)	-

Admission scores, mean ± SD					

SAPS II score	-	37.1 ± 16.4	37.7 ± 17.2	42.3 ± 19.2^$^	40.6 ± 21.2^$^

SOFA score	-	5.7 ± 3.5	5.0 ± 3.6	5.6 ± 4.3	5.7 ± 3.4

TISS-28 score	-	42.1 ± 11.3	38.4 ± 11.4	40.4 ± 13.9^$^	38 ± 14.4^$^

SOFAmax	-	6.1 ± 3.8	5.6 ± 4.3^$^	6.3 ± 4.7^$^	6.4 ± 4^$^

Mechanical ventilation					

On ICU admission (%)	193 (43.4)	184 (50.8)	150 (42)	30 (49.2)	13 (48.1)

At any time in the ICU	240 (53.9)	206 (54.1)	187 (52.4)	38 (62.3)	15 (53.6)

Duration, median and range (days)	2 (1 to 5)	2 (1 to 4)	2 (1 to 4)	5 (1 to 10)	2 (1 to 5)

Sepsis during ICU stay (%)	66 (13.9)	54 (14.2)	51 (13.4)	12 (17.9)	3 (10.7)

ICU LOS, median and range (days)	-	2 (1 to 4)	2 (1 to 4)	2 (1 to 10)	3 (1 to 8)^$^

ICU mortality rate (%)	-	-	29 (7.6)	4 (6)	6 (21.4)^$^

Hospital mortality rate (%)	-	65 (17.1)	65 (17.1)	16 (13.9)	13 (46.4)^$^

** Trauma = monotrauma without brain trauma, polytrauma without brain trauma; cardiovascular = cardiac arrest needing cardiopulmonary resuscitation prior to ICU admission, shock requiring vasopressor/inotropic drugs, chest pain, arrhythmia, cardiac failure (left, right or global); neurological = coma, stupor, vigilance disturbances, confusion, agitation, delirium, seizures, focal neurological deficit and intracranial mass effect; gastrointestinal = bleeding (gastrointestinal tract), acute abdomen, severe pancreatitis, liver failure; respiratory = acute lung injury and acute respiratory distress syndrome, acute-on-chronic respiratory failure and impaired respiratory function but less than for acute lung injury; renal = pre-renal acute renal failure, obstructive acute renal failure; haematological = haemorrhagic syndrome, disseminating intravascular coagulation; metabolic = acid-base and/or electrolyte disturbance, hypoglycaemia and hyperglycaemia.

** Renal/urinary tract, obstetric/gynaecological.

^$ ^p < 0.05 compared with initial admission.

LOS = length of stay;SD = standard deviation; SIRS = systemic inflammatory response syndrome; SAPS = simplified acute physiology score; SOFA = sequential organ failure assessment; TISS = therapeutic intervention scoring system.

Gastrointestinal surgery was the most common type of surgery performed within 24 hours of ICU admission in patients who were readmitted to the ICU more than once. Cardiovascular complications necessitating readmission were more frequent during the first readmission, whereas respiratory and gastrointestinal complications were more frequent thereafter. SAPS II scores were higher and TISS-28 scores were lower after second and third readmissions compared with the initial admission.

### Morbidity and mortality

On initial admission to the ICU, serum bilirubin concentrations, C-reactive protein (CRP) concentrations and platelet counts were similar in all patients, and creatinine concentrations, arterial lactate and leucocyte count were higher in patients who were readmitted to the ICU compared with those who were not (Table 3). The maximum concentrations of serum bilirubin, serum creatinine, leucocyte count, arterial lactate and CRP were higher in patients who were readmitted to the ICU compared with those who were not. Serum creatinine and CRP concentrations within 24 hours of initial discharge from the ICU were higher in patients who were readmitted to the ICU compared with those who were not.

**Table 3:** Laboratory parameters during intensive care unit (ICU) stay.

	No readmission (n = 2471)	Readmission (n = 381)	p value
Bilirubin (μmol/L)			
First	16 (11 to 23)	17 (11 to 25)	0.157
Max	16 (12 to 24)	19 (12 to 27)	0.009
Last	13.5 (9 to 19)	14 (9 to 21)	0.845

Creatinine (μmol/L)			
First	88 (74 to 106)	94 (79 to 120.5)	< 0.001
Max	89 (75 to 111)	99 (81 to 129)	< 0.001
Last	83 (70 to 102)	88 (72 to 119)	0.002

Leucocyte count (10^3^/μl)			
First	12.0 (9.1 to 15.5)	12.6 (9.5 to 16.6)	0.027
Max	12.5 (9.6 to 16.2)	13.4 (10.1 to 17.9)	0.002
Last	10.4 (8.2 to 13.8)	10.5 (8.1 to 14)	0.720

Platelet count (10^3^/μl)			
First	169 (127 to 224)	167 (125 to 222)	0.628
Min	159 (119 to 212)	150 (113 to 206)	0.061
Last	176 (133 to 236)	173 (130 to 242)	0.999

Lactate (mmol/L)			
First	1.7 (1.2 to 1.6)	1.9 (1.2 to 3)	0.007
Max	1.8 (1.2 to 2.8)	2 (1.3 to 3.3)	0.004
Last	0.9 (1.3 to 1.8)	1.2 (0.9 to 1.7)	0.526

C-reactive protein (mg/L)			
First	64.8 (33.4 to 102)	71.8 (34 to 113)	0.138
Max	93.5 (49.2 to 174.6)	125 (63.8 to 207.1)	< 0.001
Last	77 (38.9 to 131)	84 (40.7 to 158)	0.028

**Figure 2:**
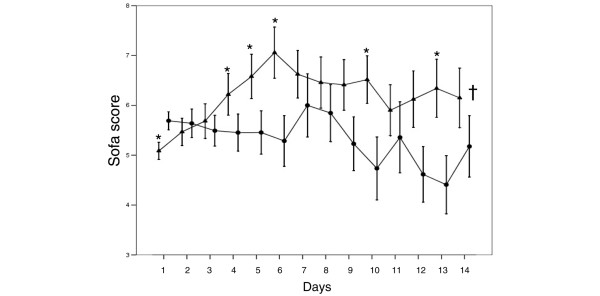
**Time course of sequential organ failure assessment (SOFA) score during the first two weeks in the intensive care unit (ICU) in patients who were readmitted to the ICU**. Closed circles = scores during the initial stay; closed triangle = score during the first readmission. *p < 0.05 compared with initial stay (Mann Whitney U test); †p < 0.05 over time (Friedmann test).

The overall incidence of sepsis syndromes was 9.1% (n = 260). Sepsis syndromes occurred more frequently during the initial admission (14.2% versus 8.3%; p = 0.001) in patients who were readmitted to the ICU. The incidence of sepsis syndromes and mechanical ventilation and the duration of mechanical ventilation were similar during initial and subsequent readmissions. In patients who were readmitted to the ICU, SOFA scores at admission were higher on initial admission to the ICU than on the first readmission; however, the SOFA scores increased steadily over the first few days of the first readmission and remained high during the first two weeks of readmission (Figure 2).

In-hospital mortality was significantly higher in patients readmitted to the ICU (17.1% versus 2.9%; p < 0.001) compared with those that were not. Patients who were readmitted to the ICU more than one week after the initial discharge from the ICU (late readmissions; n = 176) had higher in-hospital mortality rates (22.2% versus 12.7%; p < 0.001) compared with those who were readmitted within 48 hours of initial discharge (early readmission, n = 57). Readmission more than two-times to the ICU was associated with higher ICU mortality (21.4% versus 7.6%; p = 0.004) and in-hospital mortality rates (46.4% versus 17.1%; p < 0.001), and longer ICU length of stay (median = three days (range = one to eight days) versus two day(one to four days); p = 0.02) compared with the first readmission. Hospital mortality was similar for planned and unplanned readmissions (17.6% versus 15.7%; p = 0.667).

### Risk factors for readmission to the ICU

Factors associated univariately with a higher risk of ICU readmission included older age, higher SAPS II and SOFA scores on admission, admission from another hospital, unplanned admission, duration of mechanical ventilation, and higher creatinine and CRP concentrations on the day of discharge to the hospital floor (Table 4). In a multivariate analysis, age (odds ratio (OR) = 1.13 per 10 years; 95% confidence intervals (CI) = 1.03 to 1.24; p = 0.025), greater SOFAmax score (OR = 1.04 per point; 95% CI = 1.01 to 1.08; p = 0.04) and higher CRP concentration on the day of discharge to the hospital floor (OR = 1.02; 95% CI = 1.01 to 1.04; p = 0.035) were independently associated with a higher risk of readmission to the ICU.

**Table 4:** Factors associated with a higher risk of readmission to the intensive care unit (ICU).

	**Univariate**		**Multivariate**	
	**Odds ratio (95% CI)**	**p value**	**Odds ratio (95% CI)**	**p value**

Age (per 10 years)	1.14 (1.06 to 1.23)	0.001	1.13 (1.03 to 1.24)	0.025

Female gender	1.08 (0.86 to 1.36)	0.506	0.86 (0.59 to 1.24)	0.404

Source of admission				

Operating room	Reference	NA	Reference	NA

Emergency room	1.31 (0.80 to 2.15)	0.278	1.51 (0.59 to 3.84)	0.385

Other hospital	1.75 (1.17 to 2.62)	0.006	1.35 (0.60 to 3.05)	0.472

Cancer	0.82 (0.62 to 1.07)	0.148	1.05 (0.63 to 1.76)	0.845

Chronic heart failure	1.73 (0.85 to 3.49)	0.129	1.13 (0.43 to 2.94)	0.806

Chronic renal failure	1.34 (0.96 to 1.87)	0.083	1.17 (0.72 to 1.91)	0.591

Diabetes	1.24 (0.97 to 1.59)	0.093	1.47 (0.99 to 2.16)	0.054

Unplanned admissions	1.66 (1.30 to 2.12)	< 0.001	0.84 (0.42 to 1.68)	0.612

Sepsis during initial ICU stay				

No sepsis	Reference	NA	Reference	NA

Sepsis	1.46 (0.95 to 2.25)	0.083	1.18 (0.73 to 1.90)	0.494

Severe sepsis	1.44 (0.86 to 2.41)	0.171	1.04 (0.58 to 1.86)	0.901

Type of surgery				

Neurosurgery	0.97 (0.71 to 1.31)	0.822	0.97 (0.56 to 1.70)	0.923

Thoracic surgery	0.84 (0.51 to 1.39)	0.492	1.30 (0.56 to 3.06)	0.543

Cardiac surgery	0.83 (0.66 to 1.05)	0.118	0.71 (0.44 to 1.15)	0.166

Gastrointestinal	1.05 (0.80 to 1.37)	0.714	0.82 (0.56 to 1.65)	0.654

Trauma	0.86 (0.53 to 1.39)	0.548	0.79 (0.32 to 1.92)	0.601

Weekend discharge	0.79 (0.74 to 1.82)	0.175	0.84 (0.61 to 1.34)	0.575

Nocturnal discharge	0.93 (0.47 to 1.22)	0.375	0.98 (0.74 to 1.22)	0.442

Severity scores (per point)*				

SAPS 2 score**	1.02 (1.01 to 1.02)	< 0.001	1.03 (0.99 to 1.07)	0.155

SOFA score**	1.06 (1.02 to 1.09)	0.001	1.03 (0.99 to 1.07)	0.138

SOFAmax	1.06 (1.03 to 1.10)	< 0.001	1.04 (1.01 to 1.08)	0.045

Mechanical ventilation during ICU stay	1.04 (0.82 to 1.31)	0.772	1.05 (0.78 to 1.41)	0.765

Duration of mechanical ventilation (per day)	1.04 (1.01 to 1.06)	0014	1.02 (0.98 to 1.05)	0.421

Laboratory parameters on the day of initial discharge †				

Bilirubin (μmol/L)	0.98 (0.98 to 1.01)	0.558	1 (0.99 to 1.04)	0.939

Creatinine (μmol/L)	1.02 (1.01 to 1.03)	0.04	1.01 (1 to 1.03)	0089

Leucocyte count (10^3^/μl)	1.01 (0.98 to 1.03)	0.503	1.02 (0.99 to 1.05)	0.3

Platelet count (10^3^/μl)	1 (0.99 to 1.01)	0.445	1 (0.99 to 1.02)	0.543

Lactate (mmol/L)	0.94 (0.84 to 1.06)	0.308	0.95 (0.84 to 1.07)	0.413

C-reactive protein (mg/L)	1.01 (1.01 to 1.02)	0.003	1.02 (1.01 to 1.04)	0.035

Hosmer and Lemeshow Chi-squared = 11.8, p = 0.16

*Introduced sequentially in the model due to co-linearity.

**On initial admission to the ICU

†per 10 unit increase (creatinine, leucocyte count, platelet count and C-reactive protein) and per one unit increase (bilirubin and lactate)

CI = confidence interval; SAPS = simplified acute physiology score; SOFA = sequential organ failure score.

### Predictors of worse outcome in patients readmitted to the ICU

In patients who were readmitted to the ICU, the presence of cancer, chronic renal failure, gastrointestinal surgery before initial admission and greater SAPS II score were associated univariately with a higher risk of in-hospital mortality (Table 5). In a multivariate analysis with hospital mortality as the dependent variable, SAPS II (OR = 1.02 per point; 95% CI = 1.01 to 1.04; p = 0.045), chronic renal failure (OR = 2.39; 95% CI = 1.01 to 5.2; p = 0.028) and admission after gastrointestinal surgery (OR = 2.6; 95% CI = 1.17 to 5.8; p = 0.02) were independently associated with a higher risk of in-hospital death in these patients.

**Table 5:** Factors associated with a higher risk of in-hospital mortality in patients readmitted to the intensive care unit (ICU).

	**Univariate**		**Multivariate**	
	**Odds ratio (95% CI)**	**p value**	**Odds ratio (95% CI)**	**p value**

Age (per 10 years)	1.18 (0.97 to 1.44)	0.108	-	-

Female	0.98 (0.89 to 1.21)	0.205	-	-

Source of admission				

Operating room	Reference	NA	-	-

Emergency room	1.21 (0.39 to 3.79)	0.741	-	-

Other hospital	1.08 (0.42 to 2.76)	0.877	-	-

Cancer	2.21 (1.21 to 4.03)	0.010	1.69 (0.81 to 3.53)	0.161

Chronic heart failure	1.22 (0.25 to 5.89)	0.803	-	-

Cirrhosis	1.22 (0.25 to 5.89)	0.803	-	-

Chronic renal failure	2.57 (1.30 to 5.08)	0.006	2.39 (1.10 to 5.20)	0.028

Diabetes	1.30 (0.72 to 2.36)	0.380	-	-

Unplanned admissions	0.88 (0.48 to 1.60)	0.667	-	-

Sepsis during initial ICU stay				

No sepsis	Reference	NA	-	-

Sepsis	1.44 (0.60 to 3.48)	0.419	-	-

Severe sepsis	0.64 (0.18 to 2.34)	0.501	-	-

Type of surgery				

Neurosurgery	0.35 (0.12 to 1.00)	0.051	0.46 (0.14 to 1.48)	0.193

Thoracic surgery	1.42 (0.45 to 4.44)	0.553	-	-

Cardiac surgery	0.49 (0.26 to 0.92)	0.026	0.54 (0.23 to 1.25)	0.149

Gastrointestinal	3.39 (1.90 to 6.04)	< 0.001	2.60 (1.17 to 5.80)	0.020

Trauma	2.19 (0.81 to 5.94)	0.122	2.27 (0.72 to 7.18)	0.165

Time to readmission				

Within 48 hours	References	NA	Reference	NA

2 to 7 days	1.05 (0.42 to 2.66)	0.914	0.81 (0.34 to 2.26)	0.792

> 7 days	2.02 (0.81 to 5.02)	0.131	1.73 (0.69 to 4.37)	0.245

Severity scores (per point) *				

SAPS 2 score **	1.02 (1.01 to 1.03)	0.043	1.02 (1.01 to 1.04)	0.045

SOFA score **	1.04 (0.97 to 1.13)	0.276	1.07 (0.98 to 1.16)	0.163

SOFAmax	1.03 (0.96 to 1.11)	0.382	1.05 (0.97 to 1.14)	0.231

Hosmer and Lemeshow chi-squared = 7.1, p = 0.526.

* Introduced sequentially in the model due to co-linearity.

** On initial admission to the ICU.

CI = confidence interval; SAPS = simplified acute physiology score; SOFA = sequential organ failure score.

## Discussion

In this large cohort of surgical ICU patients, 13.4% of patients discharged from the ICU required readmission during the same hospitalisation. Patients who were readmitted to the ICU had a higher incidence of sepsis syndromes and comorbid conditions on initial admission to the ICU compared with those who were not readmitted. Readmission to the ICU was associated with a more than five-fold increase in hospital mortality. Older age, higher SOFAmax score and greater CRP concentrations on the day of discharge to the hospital floor were independently associated with a higher risk of readmission to the ICU.

The readmission rate in our study (13.4%) is higher than rates reported by previous authors [1,4,8,10,15]. Rosenberg and Watts [22], reported a mean readmission rate of 6% (range = 5% to 14%) in a systematic review of studies evaluating ICU readmission rates. In another recent review of 20 studies, Elliot [7] reported an average readmission rate of 7.8% (range = 0.89% to 19%). In surgical ICU patients, the readmission rates cited in the literature range between 0.89% and 9.4% [3-5,13,14,16,23,24]. Snow and colleagues [4] reported a readmission rate of 9.4%. However, this study, and others [5,25], did not exclude patients who were not at risk of readmission, that is patients who died in the ICU or who were discharged home directly from the ICU. Nishi and colleagues [5] reported a readmission rate to the surgical ICU as low as 0.89%; however, this study considered early readmissions only (within 48 hours of ICU discharge). In our study, the early readmission rate was 2% (57 of 2852). This variability in readmission rates is probably due to institutional factors [26,27] and differences in case mix [10,28,29].

In our institution, patients are not discharged from the ICU unless they are haemodynamically stable with an acceptable general condition because of the absence of intermediary care units or step-down facilities. However, this lack of intermediary units may nevertheless explain, in part, the relatively high rates of readmission, as all patients in need of vital sign monitoring are admitted directly to the ICU. The postoperative nature of the ICU may also be responsible for the higher readmission rate: about 47% of readmission episodes in our study followed surgical procedures that were scheduled in advance. With no reduction in personnel or in medical activities during the weekend or at night in the ICU, it was not surprising that nocturnal and weekend discharges had no influence on readmission rates in our cohort.

In agreement with previous studies [1-4,8-10,30], we found that cardiovascular and respiratory complications were the most common reasons for unplanned readmissions. Whether these readmissions represent early inappropriate discharges from the ICU remains a matter of speculation. However, we identified several factors that were associated with an increased risk of readmission to the ICU, including older age, higher SAPS II and SOFA scores on admission, admission from another hospital, unplanned admission, and higher creatinine and CRP concentrations on the day of discharge to the hospital floor. Similar risk factors for readmission to the ICU have been reported before [1,2,5,9,10,15,31] and may be important in risk stratification of patients discharged from the ICU. In a multivariate analysis, older age, higher SOFAmax score during the initial ICU admission, and greater CRP concentrations on the day of discharge to the hospital floor, were independently associated with a higher risk of readmission to the ICU. This finding may indicate that there was residual organ dysfunction and/or an inflammatory process that deteriorated on the hospital floor after ICU discharge resulting in subsequent readmission.

Likewise, Ho and colleagues [32] studied 1405 consecutive mixed medico-surgical ICU patients and observed that a CRP concentration that was persistently elevated during the 24 hours before ICU discharge was associated with ICU readmission. The reason for this association is uncertain and cannot be explained by the presence of sepsis or severe sepsis in our study as we adjusted for this in the multivariate analysis. CRP is an acute-phase reactant and its concentrations correlate with organ dysfunction in critically ill patients [33,34] and tend to reduce as sepsis resolves in survivors but remain elevated in non-survivors of sepsis [33,35]. High CRP concentrations have also been shown to be an independent risk factor for hospital readmission and mortality in patients with heart failure [36].

Our data confirm the association between ICU readmission and higher morbidity and mortality rates. Patients who were readmitted to the ICU in our study had a higher degree of organ dysfunction and tissue inflammation compared with those who were not readmitted. Interestingly, the first readmission episode was associated with a marked deterioration in organ function during the two weeks after readmission to the ICU compared with the initial admission. This may explain the elevated hospital mortality among readmitted patients. Previous studies have reported mortality rates of 12% to 58% in readmitted patients [3,4,8,30] with a 4- to 11-fold increase in mortality [1,10,15] compared with non-readmitted patients.

In contrast to previous studies that reported similar outcomes regardless of the time of readmission to the ICU [15,31], in our study patients who were readmitted to the ICU more than one week after the initial discharge (late readmissions) had greater in-hospital mortality rates compared with those who were readmitted within 48 hours of initial discharge (early readmissions). Nevertheless, in a multivariate analysis with hospital mortality as the dependent variable, SAPS II, the presence of chronic renal failure and admission after gastrointestinal surgery were independently associated with a higher risk of in-hospital death adjusting for time to ICU readmission. Therefore, severity of illness, comorbidities and surgical interventions, rather than time to readmission, are the major determinants of prognosis in patients who are readmitted to the ICU.

Our study has some limitations. First, the multivariate approach is limited by the variables included in the analysis; therefore, unmeasured variables may have influenced the results. However, we included a large number of relevant data including parameters of organ failure and markers of tissue inflammation on the day of initial discharge from the ICU. Second, due to the observational nature of our study, we could not determine whether readmissions were appropriate or not. However, we identified some risk factors for readmission that may be useful in risk stratification of patients discharged from the ICU. Prospective studies with predefined criteria based on risk factors reported from observational studies such as the present are warranted. Finally, our results may not apply to other ICUs with a different case-mix such as medical or mixed medico-surgical ICU patients. Nevertheless, our data provide important insights into the incidence of, outcome from and risk factors for readmission to a surgical ICU.

## Conclusion

In this large cohort of surgical ICU patients, 13.4% of patients discharged from the ICU required readmission during the same hospitalisation. Readmission to the ICU was associated with a more than five-fold increase in hospital mortality. Older age, higher SOFAmax score and greater CRP concentrations on the day of discharge to the hospital floor were independently associated with a higher risk of readmission to the ICU.

## Key messages

• In this large cohort of surgical ICU patients, 13.4% of patients discharged from the ICU required readmission during the same hospitalisation.

• Readmission to the ICU was associated with a more than five-fold increase in hospital mortality.

• Older age, higher SOFAmax score and greater CRP concentrations on the day of discharge to the hospital floor were independently associated with a higher risk of readmission to the ICU.

## Abbreviations

CI: confidence interval; CRP: C-reactive protein; ICU: intensive care unit; OR: odds ratio; SAPS: simplified acute physiology score; SD: standard deviation; SOFA: sequential organ failure assessment; TISS: therapeutic intervention scoring system.

## Competing interests

The authors declare that they have no competing interests.

## Authors' contributions

All authors participated in the design of the study. AK and YS contributed to the data collection and statistical analysis. AK, FC and YS drafted the manuscript. KR, US, JG and RK revised the article. All authors read and approved the final manuscript.
